# The Psychological Impact of Male Infertility: A Narrative Review

**DOI:** 10.7759/cureus.89453

**Published:** 2025-08-05

**Authors:** Swagata Sahoo, Aditi Das, Rojalin Dash, Anasuya Behera, Nilam Mishra, Karishma Bal

**Affiliations:** 1 Obstetrics and Gynaecological Nursing, Kalinga Institute of Nursing Sciences, Kalinga Institute of Industrial Technology, Deemed to be University (KIIT-DU), Bhubaneswar, IND; 2 Obstetrics and Nursing, Kalinga Institute of Nursing Sciences, Kalinga Institute of Industrial Technology, Deemed to be University (KIIT-DU), Bhubaneswar, IND; 3 Obstetrics and Gynaecology, Kalinga Institute of Nursing Sciences, Kalinga Institute of Industrial Technology, Deemed to be University (KIIT-DU), Bhubaneswar, IND

**Keywords:** anxiety, depression, male infertility, masculine identity, psychological impact

## Abstract

Male infertility is a major health concern worldwide. While biological causes are well understood, the psychological aspects receive less focus. This gap is evident in clinical practice and research, where emotional, social, and mental health issues linked to male infertility are often neglected or inadequately managed. This review aims to highlight the psychological effects of male infertility, emphasizing mental health, emotional well-being, and the sociocultural factors influencing men's experiences. A comprehensive literature search was conducted using PubMed and Web of Science, encompassing English-language studies published between 2010 and 2025. Of the 118 articles identified, 36 met the inclusion criteria based on methodological rigor and relevance. These studies included observational research, clinical trials, qualitative analyses, and systematic reviews that examined psychological outcomes among men diagnosed with infertility. The findings consistently indicate that male infertility is associated with significant psychological distress, including depression, anxiety, diminished self-esteem, and disruptions to masculine identity. Sociocultural expectations that equate fertility with manhood often exacerbate these emotional burdens, especially in patriarchal contexts where infertility is stigmatized or incorrectly attributed to external or spiritual causes. Many men report feelings of emotional suppression, isolation, and reluctance to seek support, which are further complicated by sexual dysfunction and relationship strain. Despite these challenges, psychological support for men remains limited within infertility services, which primarily focus on female partners. This review emphasizes the need for a more inclusive, gender-sensitive, and psychologically informed approach to the care of male infertility. Recognizing male infertility as a complex psychological experience is essential for improving outcomes and promoting holistic reproductive health.

## Introduction and background

Infertility represents a substantial public health issue impacting individuals, couples, and communities on a global scale. Epidemiological data suggest that approximately one in six individuals of reproductive age will experience infertility at some point during their lifetime, underscoring its pervasive nature and significant societal implications [1].

Infertility is a disease of the male or female reproductive system defined by the failure to achieve a pregnancy after 12 months or more of regular unprotected sexual intercourse. Infertility is typically classified as either primary, when pregnancy has never been achieved, or secondary, when an individual is unable to conceive again after a prior pregnancy [2]. Infertility is a complex issue with a variety of potential causes that may arise from male factors, female factors, or a combination of both. According to the World Health Organization (WHO), male infertility is defined as the inability of a man to cause pregnancy in a fertile female after 12 months of regular unprotected intercourse [1].

In the male reproductive system, infertility is most commonly attributed to issues with semen ejection, low sperm concentration or absence of sperm, and abnormalities in sperm morphology and motility [1,3]. Male factors are solely responsible in 20-30% of cases and contribute to approximately 50% of infertility cases overall [4,5].

Almost 15% of couples, which is approximately 48.5 million couples globally, deal with male infertility. Unfortunately, there is a lack of detailed information about this problem, especially in areas that lack resources [4,6]. Variations between regions and insufficient reporting contribute to the absence of precise epidemiological data regarding male reproductive health [4].

Fertility care encompasses prevention, diagnosis, and treatment of infertility. However, equitable access to fertility services remains limited, particularly in low- and middle-income countries (LMICs), where such services are often not included in universal health coverage benefit packages [1,6]. A recent WHO report found a global lifetime prevalence of infertility at 17.5%, with 17.8% in high-income countries and 16.5% in LMICs, reinforcing the urgent need for accessible, high-quality fertility care [1].

Male infertility, although equally important, has received less focus than female infertility in both clinical practice and research due to sociocultural biases that predominantly associate infertility with women [6,7]. Common causes of male infertility include issues with spermatogenesis, hormonal imbalances, anatomical blockages, and poor semen quality, especially abnormalities in sperm shape, concentration, and movement [4,5,8]. However, these biological factors are only part of a more complex story. The emotional and psychological aspects of male infertility are often understudied and overlooked, especially in patriarchal societies where fertility is closely tied to perceptions of masculinity and social identity [9-11].

The inability to attain biological paternity can exert a profound psychological toll on affected men, frequently resulting in significant emotional distress. An increasing body of evidence has demonstrated associations between male infertility and various adverse mental health outcomes, including heightened levels of anxiety, depressive symptoms, feelings of guilt, perceived inadequacy, diminished self-esteem, and social isolation [12-15]. These psychological burdens are often exacerbated by sociocultural stigma, the invasive nature of diagnostic and therapeutic interventions, and repeated failures of assisted reproductive technologies (ART) [11,16,17]. Furthermore, the prolonged uncertainty and cyclical hope and disappointment accompanying fertility treatments can further intensify psychological strain [13,18].

In addition to emotional and cognitive impacts, male infertility can influence sexual health. Prolonged periods of infertility are correlated with an increased risk of sexual dysfunction, including erectile dysfunction, reduced libido, and performance anxiety, which may further impair quality of life and strain interpersonal relationships [15,17]. The duration of infertility and sexual dysfunction indicates a cumulative effect over time. Such challenges are frequently internalized, as many men are reluctant to express emotional vulnerability due to prevailing gender norms that discourage open discussion of mental health or reproductive difficulties [9,10,19].

Compounding this issue, psychosocial support mechanisms for men undergoing fertility treatments are often inadequate or absent. Men typically receive less emotional and psychological support during treatment compared to their female partners, despite experiencing comparable levels of stress and emotional turmoil [12,14,19]. This imbalance in psychosocial care not only marginalizes the male experience but may also negatively impact treatment adherence, marital satisfaction, and overall well-being [13,16].

While there is a growing acknowledgment of the emotional consequences of infertility, the psychological experiences of men remain insufficiently represented in discussions and care related to reproductive health. Male infertility is often viewed primarily through a biomedical framework, resulting in limited focus on its psychosocial effects. A comprehensive understanding of male infertility requires the integration of both biological and psychological factors. This review focuses on the psychological effects of male infertility, highlighting how it can impact mental health, emotional well-being, and social influences. By synthesizing current evidence across clinical, psychological, and sociocultural domains, this review aims to identify key knowledge gaps, challenge prevailing assumptions, and advocate for more inclusive, gender-sensitive frameworks within fertility care and mental health support systems.

## Review

Search methodology

This search methodology review followed a comprehensive and structured approach to identify relevant literature. The review utilized two commonly used electronic databases: PubMed and Web of Science. Search terms included ("male infertility" OR "male sub-fertility") AND ("psychological impact" OR "mental health" OR "emotional well-being" OR "psychological effects") along with relevant keywords, combined using Boolean operators to refine and optimize search results.

This narrative review examines studies investigating the psychological impact of male infertility. The included research involved men diagnosed with infertility, exploring emotional and mental health outcomes such as depression, anxiety, stress, low self-esteem, and relationship challenges. Eligible studies comprised observational research, clinical trials, qualitative analyses, and reviews published in English from 2010 to 2025. A comprehensive search identified a total of 118 articles, of which 36 met the inclusion criteria based on relevance, methodological quality, and rigor. These studies provide insights into the complex psychological burden experienced by men facing infertility and highlight factors influencing mental well-being during diagnosis, treatment, and coping processes.

Psychological impact of male infertility

Male infertility is not only a biological issue but also a significant psychological stressor that affects emotional well-being and interpersonal relationships. Men experiencing infertility often face heightened psychological distress, including depression, anxiety, lowered self-esteem, and identity crises. This highlights the need for a holistic approach that addresses both the biological and emotional aspects of infertility.

Psychological distress and mental health outcomes

Men facing infertility often experience psychological symptoms like depression and anxiety. Depression, more so than anxiety, is directly linked to low semen quality, establishing a clear feedback loop between mental health and reproductive function [20]. Infertility in men often comes with emotional distress, such as feelings of grief, isolation, and hopelessness [21]. Longer periods of infertility tend to worsen mental health, with increasing psychological stress and decreased sexual functioning over time, indicating a cumulative effect [15]. In a national study, men undergoing fertility treatment reported substantial declines in overall quality of life and increased psychological distress, especially during invasive treatment stages [22].

Masculinity and identity

Infertility presents a significant challenge to traditional concepts of masculinity. Cultural and social expectations often associate reproductive capability with male identity, leading to the perception of infertility as a failure in that identity [23]. The internal struggle can result in feelings of shame, self-doubt, and a tendency to conceal the experience of infertility. For many men, this issue profoundly undermines established notions of masculinity, leading to a deep sense of inadequacy and diminished self-worth. Fertility is frequently regarded as a fundamental aspect of male identity, making its loss a substantial psychological and emotional burden [21]. Infertility constitutes more than a mere medical condition; it poses significant challenges to one's sense of identity, masculinity, and future well-being. This experience can exacerbate feelings of anxiety, depression, and emotional distress [24].

Sexual function and relationship stress

Male infertility can negatively affect sexual health and satisfaction. The diagnosis and treatment process itself may lead to reduced libido, erectile dysfunction, and performance anxiety, creating a cyclical pattern of sexual and emotional difficulties [25]. Infertile men often experience poorer sexual function and higher levels of psychological distress than fertile men [26].

Cultural and social contexts

Cultural context significantly influences the psychological effects of male infertility. In environments where childbearing is central to male social identity, men may experience heightened stigma and societal pressure. Socio-demographic factors such as lower income, limited education, and adherence to traditional gender roles are associated with poorer psychological outcomes [27]. In these contexts, the need for compassionate genetic counseling and emotional support becomes essential [28].

Lack of psychological support

Many men experience significant psychological stress during infertility diagnosis and treatment, yet they often receive insufficient emotional or psychological support. Counseling tends to prioritize women, leaving male partners overlooked within reproductive healthcare settings [29]. Many men hide their feelings or avoid discussing their experiences, which can increase their risk of facing mental health challenges [10].

Psychosocial and relational stress in male infertility

Male infertility often brings about significant psychological stress, including feelings of inadequacy, diminished self-worth, and emotional isolation. These stressors are deeply rooted in sociocultural expectations surrounding masculinity and reproductive capability, which amplify feelings of shame and the mental burden experienced by affected men [30,31]. Many individuals dealing with infertility report heightened levels of anxiety, depression, and lowered self-esteem, which can lead to strained sexual relationships and reduced intimacy [31,32].

The psychological challenges are further exacerbated during treatment processes, such as intracytoplasmic sperm injection (ICSI), where men may feel marginalized or underrepresented in the clinical experience [31]. The implications of male infertility extend beyond individual distress to impact relational dynamics as well. Research indicates that infertility can lead to increased marital strain, communication breakdown, and emotional distance. However, some couples find that navigating these challenges together can strengthen their bond [32,33]. While social support is vital, it does not always alleviate the negative effects on marital satisfaction, especially when external pressure or perceived blame is involved [33,34].

Support mechanisms and perceived relationship quality

The perceived quality of relationships among infertile couples can vary considerably depending on whether the infertility is attributable to male or female factors. Notably, males in relationships impacted by female infertility report significantly lower relationship quality compared to those in male-factor cases, potentially due to differing emotional burdens and coping mechanisms [32]. This indicates that male infertility, while psychologically demanding, may not uniformly diminish relationship satisfaction when mutual support is present.

Social and familial support networks play a complex role; while they may provide emotional relief, they can also exacerbate societal pressures concerning masculinity and fatherhood [33]. Counseling that addresses the emotional needs of the male partner, encourages open communication, and promotes joint coping strategies has been demonstrated to enhance dyadic adjustment and overall relationship stability [30,31,35].

Social stigma and superstition of male infertility

Male infertility bears social stigma across diverse cultures, particularly in patriarchal societies where fertility is intrinsically linked to masculinity. The inability to conceive is frequently perceived as a challenge to male identity, engendering feelings of inadequacy, diminished self-esteem, and emotional isolation [36]. In numerous instances, infertility is automatically ascribed to the female partner, thereby perpetuating silence concerning male reproductive issues [36]. Superstitions also substantially influence societal beliefs. In various communities, male infertility is attributed to spiritual causes such as curses, divine punishment, or the evil eye [37]. Such beliefs discourage men from pursuing medical intervention and often lead them toward unscientific or traditional healing practices, thereby delaying effective treatment [38]. Moreover, stigma affects healthcare behavior; men frequently avoid testing and fertility assessments due to apprehension of being perceived as weak or emasculated [36,39]. Even when they undertake treatment, they may experience exclusion, as fertility care systems tend to prioritize the female partner [39]. The gap in supportive care makes things even more challenging for men dealing with infertility (Table 1).

**Table 1:** Features of the included review articles QoL: quality of life

Author	Year	Key Findings	Conclusion
Agarwal et al. [4]	2015	Explored global perspectives on male infertility.	Male infertility is multifactorial and varies across cultures.
Agarwal et al. [5]	2021	Reviewed causes, diagnosis, and treatment of male infertility.	Multidisciplinary care is essential for effective management.
Purkayastha & Sharma [6]	2020	Analyzed prevalence and determinants of primary infertility in India.	Socioeconomic and demographic factors are significant.
Clark et al. [7]	2025	Discussed male sexual disorders, including infertility.	Infertility and low libido are closely linked to mental health issues.
Miner et al. [9]	2018	Explored masculinity and mental health among infertile men and cancer patients.	Masculinity norms affect support-seeking and mental health.
Dooley et al. [10]	2014	Studied the psychological impact of infertility on male partners.	Fertility treatment can lead to significant distress.
Braverman et al. [11]	2024	Summarized recent global research on mental health and infertility.	Infertility increases risk of anxiety and depression.
Sharma & Shrivastava [12]	2022	Highlighted psychological issues related to infertility.	Psychological support is essential.
Wischmann [13]	2024	Reviewed psychological aspects of infertility.	Mental health must be addressed in infertility care.
Choudhary et al. [14]	2025	Discussed mental health integration in infertility.	Infertility treatment should include psychological care.
Dong et al. [15]	2022	Studied infertility duration and its effect on sexual/mental health.	Longer infertility is linked to worse outcomes.
Reisi et al. [16]	2024	Evaluated couple collaboration and well-being.	Cooperation improves psychological outcomes.
Xue et al. [17]	2023	Investigated family dignity interventions.	Positive effects on mental health and satisfaction.
Klemetti et al. [18]	2010	National survey of infertility and mental disorders.	Infertile individuals are at higher risk of mental health issues.
Fernández-Zapata & Cardona-Maya [19]	2023	Editorial on mental health in male infertility.	Calls for prioritizing male mental health.
Zhang et al. [20]	2024	Investigated mental health and semen quality.	Depression negatively impacts semen quality more than anxiety.
Drewitt & Marczak [21]	2024	Metaethnographic review of men's infertility experiences.	Infertility deeply affects identity and mental well-being.
Cegar et al. [22]	2023	Assessed quality of life in Serbian men undergoing infertility treatment.	High psychological distress observed.
Wischmann & Thorn [23]	2013	Mixed-methods study on the meaning of infertility in men.	Stigma and identity issues are significant.
Kaltsas et al. [24]	2025	Reviewed infertility and life expectancy.	Infertility may be a marker of broader health risks.
Biggs et al. [25]	2024	Systematic review on psychological consequences.	Infertility causes serious emotional distress.
Sahin et al. [26]	2025	Compared infertile and fertile men's mental and sexual health.	Infertility linked to poorer outcomes.
Koochaksaraei et al. [27]	2016	Studied mental health predictors in Iranian infertile men.	Socio-demographic factors influence mental health.
Bereketolu et al. [28]	2022	Reviewed genetic causes and need for counseling.	Advocated for genetic counseling in infertility.
Petok [29]	2015	Critiqued the lack of male infertility counseling.	Men are often overlooked in fertility counseling.
Jain & Khan [30]	2025	Reviewed psychosocial challenges.	Infertility causes stress, isolation, and relationship tension.
Wu et al. [31]	2023	Systematic review of men's challenges in treatment.	Emphasizes the need for holistic care in male infertility.
JamaliGandomani et al. [32]	2022	Studied infertility’s effect on relationship quality.	Infertility negatively affects couple satisfaction.
Tabassum et al. [33]	2023	Explored stress and social support's effect on marital satisfaction.	Social support mitigates stress impacts.
Martins et al. [34]	2014	Investigated perceived support among infertile couples.	Support improves resilience and well-being.
Dourou et al. [35]	2023	Assessed QoL in infertile couples.	Infertility harms the overall quality of life.
Ergin et al. [36]	2018	Infertility is associated with significant social stigma and negative familial attitudes, particularly for women in traditional settings.	Social and familial pressures contribute to emotional distress in individuals dealing with infertility.
Hanna & Gough [37]	2019	Male infertility affects identity and masculinity; men often feel marginalized in infertility discourse and healthcare settings.	Recognizing male perspectives is vital to providing comprehensive and empathetic infertility support.
Peterson et al. [38]	2025	Infertility stigma is linked to more depressive symptoms; openness is linked to better mental health and meaning in life.	Reducing stigma and promoting openness may improve well-being in people with infertility.
Abbasi et al. [39]	2025	Meta-ethnographic synthesis reveals recurring emotional themes: shame, isolation, and resilience among infertile men across various cultures.	Infertile men can "survive and thrive" when emotional challenges are acknowledged and psychosocial support is offered.

Recommendations

Psychological support should be acknowledged as a vital component of male infertility care, rather than viewed as an auxiliary concern. It is essential to implement routine mental health screenings and provide access to specialized counseling in fertility clinics to effectively address the high prevalence of anxiety, depression, and emotional distress experienced by men undergoing infertility evaluations and treatments.

Equally important is the need to reduce the stigma associated with male infertility. Public health strategies should focus on challenging societal and cultural norms that associate masculinity with fertility, thereby fostering a more open dialogue regarding male reproductive health. Tailored educational initiatives can play a significant role in improving help-seeking behaviors and alleviating the feelings of isolation commonly reported by affected individuals.

Future research should prioritize longitudinal and culturally diverse studies to gain a comprehensive understanding of the evolving psychological impacts of infertility over time. The development and validation of male-specific, evidence-based psychosocial interventions will enhance clinical outcomes. Moreover, integrating couple-centered approaches that strengthen communication and emotional support between partners is essential within care models.

Training programs for healthcare professionals must emphasize the psychosocial dimensions of infertility, ensuring that providers are equipped with the skills necessary to deliver empathetic and gender-sensitive care. Additionally, addressing sexual dysfunction, which is closely linked to emotional well-being, should form part of a holistic treatment plan.

**Figure 1:**
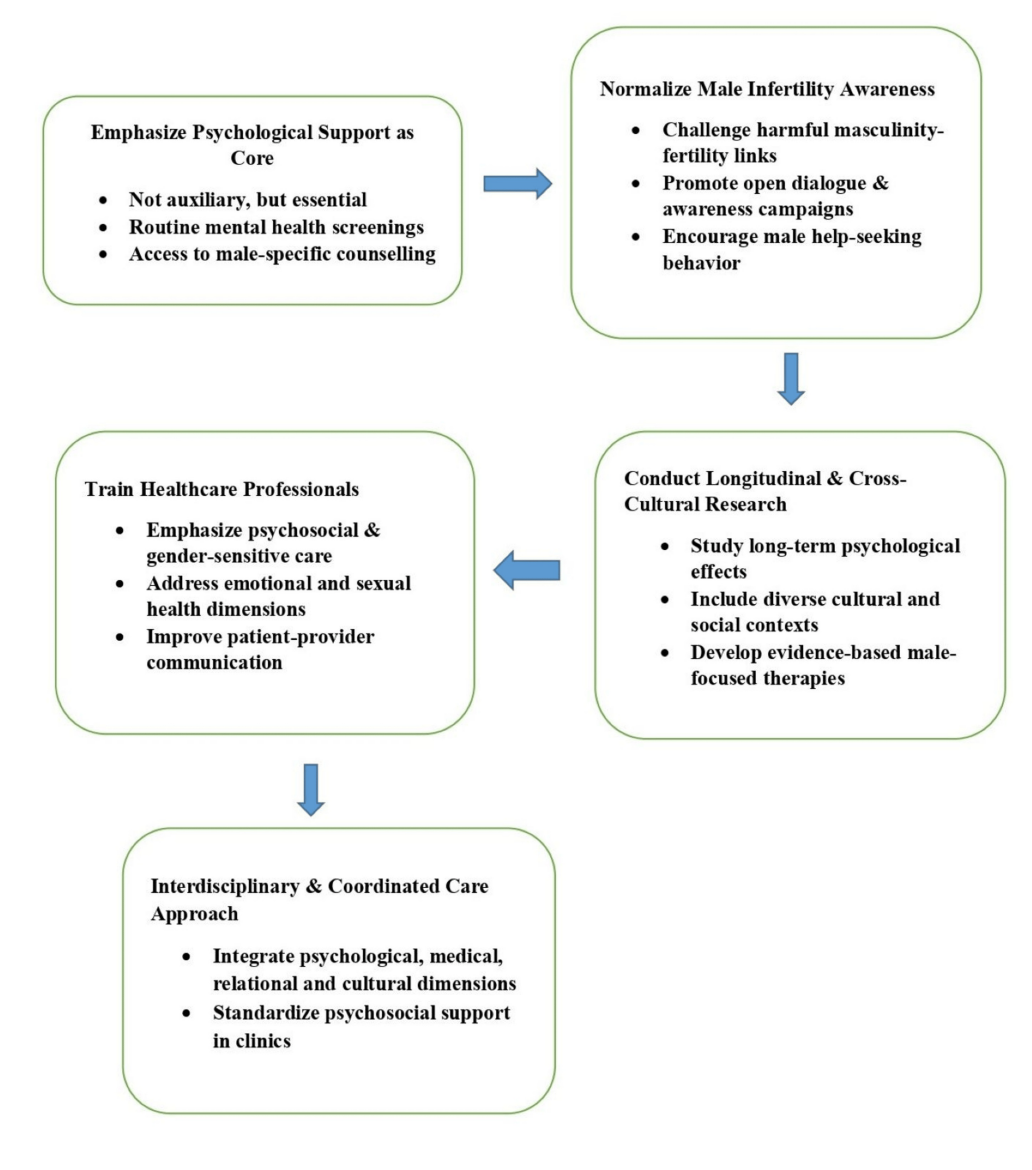
Recommendations for future implementation Image created by the authors

A coordinated and interdisciplinary approach is critical to systematically and comprehensively address the psychological needs of men experiencing infertility in both research endeavors and clinical practice (Figure 1).

## Conclusions

Male infertility is a multifaceted health concern that carries significant psychological ramifications extending beyond reproductive functionality. The emotional burden associated with this condition is often intensified by societal stigma, cultural expectations, and insufficient psychosocial support, all of which can profoundly impact mental well-being, identity, sexual health, and relationship dynamics. Despite a growing body of evidence that underscores these challenges, the experiences of men in this context remain largely underrepresented in clinical practice and research.

Addressing the psychological impact of male infertility requires a more inclusive and gender-sensitive approach in healthcare. This includes the integration of mental health services within fertility treatment frameworks, the encouragement of open discourse on the topic, and the development of culturally informed interventions designed to support affected individuals and couples. Embracing a more holistic perspective, one that acknowledges the emotional, relational, and social dimensions of infertility, will not only enhance patient outcomes but also foster greater equity in reproductive healthcare.
